# Nationalizing Operational Research Capacity Building: Necessity or Luxury?

**DOI:** 10.5334/aogh.3056

**Published:** 2020-10-20

**Authors:** Rony Zachariah, Mohammed Khogali, Ajay M. V. Kumar, Anthony D. Harries, John C. Reeder

**Affiliations:** 1 UNICEF, UNDP, WORLD BANK, WHO, Special Programme for Research and Training in Tropical Disease (TDR), World Health Organisation, Geneva, CH; 2 International Union Against Tuberculosis and Lung Disease, Paris, FR; 3 International Union Against Tuberculosis and Lung Disease, South-East Asia Office, New Delhi, IN; 4 Yenepoya Medical College, Yenepoya, Mangaluru, IN; 5 London School of Hygiene and Tropical Medicine, London, UK

“All nations should become consumers and producers of research knowledge.”

This was one of the main messages of the 2013 World Health Report titled “Research for Universal Health Coverage [1].” To make this goal a reality, research capacity needs to be built in public health programmes, close to the demand for health services.

In many low- and middle-income countries, the health system is overwhelmed with high patient load and obliged to deal with multiple diseases, all within a framework of limited human and financial resources. It is precisely in these contexts that public health programmes need to embrace a culture of inquiry, to understand what works and what does not work and find practical solutions to problems. Failure to do so can result in the health system simply continuing to deliver services without any knowledge about whether these services are effective in improving clinical and programme outcomes, whether all those in need of services, particularly the vulnerable, are able to access them and whether drugs that are being given to patients are actually working. Operational research can help to answer these questions. In the era of the Sustainable Development Goals, the answers to such questions are critical in strengthening the health systems that are necessary to achieve Universal Health Coverage [2].

A practical definition of operational research is the “search for knowledge on interventions, strategies and tools that can improve the performance of health services including quality and coverage [3].” Put simply, it is about doing better by examining the nuts and bolts of the health system, detecting problems, and building the science of solutions. There are many direct parallels between the goals, approaches, and outputs of operational research and those of implementation science, but operational research such as implementation science is generated and led from identified constraints to health care delivery. Implementation science could be seen as being broader and to do with “scalability.” However, in our opinion, there is no strict line between the two. Health systems should embrace this process as part of a continuing culture of health system accountability.

The main benefits of country-focussed operational research include: 1) improving the performance of the national health system, 2) assessing the feasibility and effectiveness of new strategies and/or interventions in specific settings and populations, and 3) informing decision making for changing policy and/or practice.

Indeed, such research has been linked to changes in and/or reinforcement of policy and program implementation [10], a synergistic benefit of conducting operational research within the health system is improved data collection, monitoring, usage and feedback [4]. Furthermore, operational research programs could be framed as building sustainable human resource capacity through a cycle of human resource development that involves “Train, Embed, Sustain, and Enable” of health staff within disease control programs and institutions.

A number of elements are needed to build and sustain operational research capacity at national level, including: building a critical mass of trained operational researchers who are embedded and retained within programmes and who train others; having a mechanism to define and prioritize country-relevant research; making sure there is engagement and buy-in of decision makers; and integrating these activities as part of country action plans and budgets. Effective dissemination including publishing in peer-reviewed journals is also needed [5]. The latter serves as a quality control mechanism and is a recognized standard in medicine [6].

The driving principle for training at country level is that it must be practical, be able to provide hands-on mentorship, and be accompanied with milestones and targets so that it is output-oriented. One proven model that encompasses these aspects is the Structured Operational Research and Training Initiative (SORT IT) [7]. It is a global partnership coordinated by the Special Programme for Research and Training for Tropical Diseases (TDR) and implemented with various partners. Aimed at making countries data rich, information rich, and action rich [8], this model has been expanded to 91 countries, trained close to 800 participants, and resulted in hundreds of peer-reviewed papers [9]. Importantly, nearly 70% of the research has contributed to a change in policy and or practice [10].

There are many examples of countries that have adapted and successfully nationalized the SORT IT model. Examples include: Kenya, where the focus was on tuberculosis and neglected tropical diseases [11]; Sierra Leone and Liberia, on the Ebola virus disease outbreak and health systems recovery [12]; Pakistan, on embracing various public health problems [13]; Armenia [14] and Ukraine [15], on access to health care among vulnerable populations; India, on tuberculosis and other training innovations [16,17]; and the United Kingdom, on health protection issues [18].

Rwanda has also adapted the SORT IT model using a deliverable-driven and learning-by-doing pedagogy. This experience was described previously as an Intermediate Operational Research Training Programme [19]. In this special supplement of the *Annals of Global Health*, several new studies are presented from Rwanda focusing on new thematic areas. These include child development, neonatology, integrating oncology services with non-communicable diseases, cervical cancer, unintentional injuries in children, registries for rheumatic heart disease, and drug stock-outs for non-communicable diseases. This is a compendium of research on public health issues that are important and useful to the Rwandan health system.

In conclusion, any country and health system striving to achieve Universal Health Coverage and excellence must be able to generate and use locally available evidence in a timely manner. This must be considered a necessity and not a luxury. Ways forward in mobilizing resources to build, sustain, and expand operational research initiatives at country level are urgently needed.
